# Pitfalls preventing bone union with EXOGEN Low-Intensity Pulsed Ultrasound

**DOI:** 10.1051/sicotj/2022012

**Published:** 2022-04-15

**Authors:** Luke D. Hughes, Jamal Khudr, Edward Gee, Anand Pillai

**Affiliations:** 1 Stepping Hill Hospital, Poplar Grove Hazel Grove, Stockport SK2 7JE UK; 2 Royal Liverpool Hospital Prescot St. Liverpool L7 8XP UK; 3 Salford Royal Hospital Stott Lane, Salford M6 8HD UK; 4 Wythenshawe Hospital Southmoor Rd Wythenshawe, Manchester M23 9LT UK

**Keywords:** Non-union, Non-invasive, Protocol, Compliance, Cost

## Abstract

*Objectives*: To evaluate the efficacy of EXOGEN in achieving union and common pitfalls in its use within the Manchester Foundation Trust (MFT) and Salford Royal Foundation Trust (SRFT). *Method*: Patients receiving EXOGEN therapy between 01/01/2017 and 31/12/2019 at hospitals within MFT and SRFT were identified using EXOGEN logbooks and hospital IT systems. An equal number of patients were included from both sites. Data were retrospectively collected from clinical documents detailing clinical presentation comorbidities, and radiographic images, determining the radiological union post EXOGEN therapy. In addition, local practices were observed and compared to EXOGEN’s standardized guidance for clinicians. *Results*: Fifty-eight patients were included in the primary review, with 9 subsequently excluded based on insufficient clinical data. 47% of patients achieved radiological union following completion of EXOGEN therapy. Outcomes of the 23 patients with persistent non-union were as follows – 18 were referred for revision surgery, 5 were prescribed further EXOGEN therapy, 2 refused or were unfit for further intervention, and 1 did not have a plan documented. No significant baseline differences were present in both outcome groups. However, at MFT and SRFT, rates of union with EXOGEN are below that previously published in the literature. *Conclusion*: EXOGEN has proven successful in facilitating union in established cases of non-union without the risk and cost associated with revision surgery. Centre outcome differences may be explained by failure to educate clinicians and patients on the correct use of the EXOGEN device, failure to standardize follow-up or monitor compliance, and must be addressed to improve current services.

## Introduction

Fracture non-union can have devastating consequences for the patient. Defined as a permanent failure of bone healing, diagnosis is typically made after 6–8 months [1]. Risk factors for non-union are well described in the literature. These include the location of the injury, with tibial fracture posing the greatest risk [2]; the magnitude of the injury [3], open fractures and soft tissue compromise [2, 4, 5]; contamination and infection; poor vascularity [6]; smoking [7]; malnutrition; diabetes [8]; osteoporosis [9]; inadequate stabilization and movement at the fracture site [10, 11]; and poor bone contact [12].

In order to facilitate fracture healing, it is important to ensure there is no soft tissue interposition at the fracture site, that the bone fragments are well aligned, with no distraction, while accommodating for any bone loss through grafting. Respect the soft tissues, as excessive intra-operative periosteal stripping will further compromise the vascularity and contribute to non-union.

Rates for non-union vary widely in the literature. A more recent study presenting figures from a population of over 4 million gave an overall non-union per fracture of 1.9%; 1.5% in women, and 2.3% in men [2]. Patients often complain of persistent pain and instability, with radiological imaging demonstrating a residual fracture gap. Further intervention may be required to facilitate bone healing and achieve union in this setting. This has traditionally involved revision surgery and bone grafting, exposing the patient to additional risk and incurring an estimated cost of between £7,000 and £79,000 [13–16]. As such, if a non-invasive, low-cost alternative is proposed, this should be fully investigated.

A range of EXOGEN low-intensity pulsed ultrasound (LIPUS) bone healing devices has been developed by Smith and Nephew, with the EXOGEN 4000+ intended for use in patients with fracture non-unions. Costing £2,562.50, it is significantly cheaper than the expenses quoted for revision surgeries. The device delivers a minimum of 191 × 20-minute treatments (over 6 months treatment). Lightweight and durable, EXOGEN is designed for easy application with a simple user interface. It allows a patient to take control of their treatment, delivering therapy in the comfort of their own home.

The transducer produces low-frequency pulsed ultrasound waves, which act upon osteoprogenitor cells, manipulating the processes of mechano-transduction. It can stimulate new bone formation and facilitate bone union without further surgery. The use of ultrasound waves upregulates mRNA within osteoprogenitor cells, producing more Cbfa1/Rnx2 and osteocalcin necessary for osteogenesis [17]. There is also an associated increased production of Prostaglandin E2 and nitric oxide, which help facilitate the tracking of inflammatory cells to the injury site [18, 19]. This happens by inducing local vasodilatation, increasing blood flow, and increasing vascular permeability. When combined with nitric oxide’s innate antimicrobial properties, their use to regulate inflammatory pathways stimulates osteoblast differentiation and proliferation, thus increasing new bone formation, accelerating bone union, and subsequent remodeling [18, 20–22].

The National Institute for Health and Care Excellence (NICE) has reviewed the available evidence on the efficacy of LIPUS in facilitating the healing of fracture non-unions. In doing so, NICE has concluded that LIPUS is safe to use despite acknowledging that the current evidence on efficacy is inadequate in quality. As such NICE recommends that LIPUS can only be used with special arrangements for clinical governance, consent, and audit or research. Currently, clinicians who wish to use a LIPUS system must consult with their clinical governance leads, fully inform the patient about the uncertain efficacy and support a shared decision-making process. Furthermore, clinicians are encouraged to audit outcomes [23].

With a looming uncertainty around the efficacy of LIPUS and a highlighted need for further evidence by NICE, we endeavored on this study to evaluate the efficacy of EXOGEN for the management of non-union at Manchester Foundation Trust (MFT) and Salford Royal Foundation Trust (SRFT). We hope to explore our experienced rates of union and compare them to those previously published. We also hope to share our experience of working with this new technology, identify difficulties, and raise awareness of any pitfalls to improve clinical practice, increase union rates and improve cost-effectiveness.

## Methodology

### Objective

This project is a retrospective case series aimed at evaluating the efficacy of EXOGEN in achieving union and common pitfalls in its use within the MFT and SRFT.

### Study design and protocol

All patients presenting to hospitals within the MFT (including Altrincham, Trafford, Wythenshawe, and Manchester Royal Infirmary) and the SRFT with non-union and managed using the EXOGEN LIPUS system between 01/01/2017 and 31/12/2019 were identified using the EXOGEN patient logs, hospital IT systems, and patient notes.

### Patients

A minimum therapy duration of 120 days was determined prior to the analysis of outcomes. All patients presenting to MFT were analyzed, while a patient sample of equal size was selected from the SRFT cohort using a random number generator. Exclusion criteria included any patients with insufficient clinical documentation and/or radiographs.

**Figure 1:**
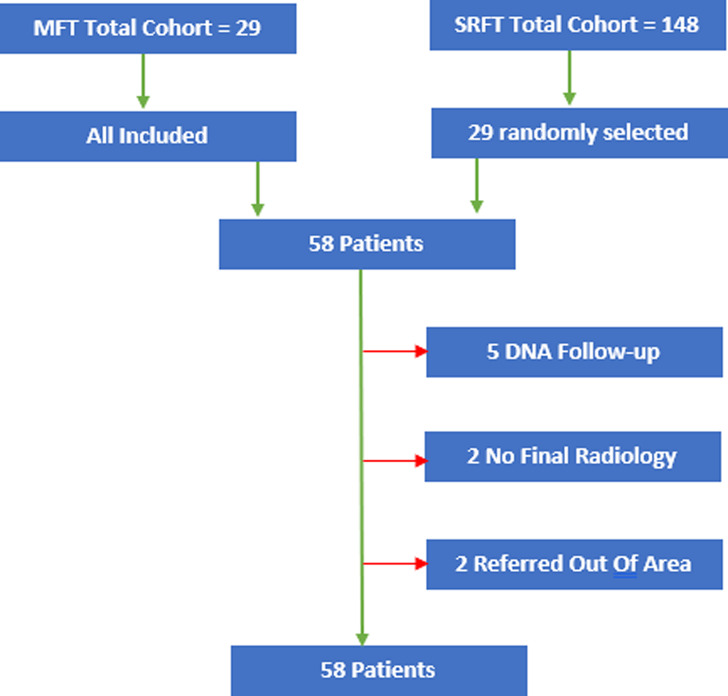
Patient sample flow chart, showing initial recruitment, exclusions with reasons specified, and final included patient sample.

A total of 29 patients received EXOGEN LIPUS therapy to manage non-union at the MFT between 01/01/17 and 31/12/19. A sample of 29 patients was selected at random from a total cohort of 148 patients presenting to the SRFT over the same time period. Nine patients were excluded (5 failed to attend follow-up appointments, 2 had no final radiological images, 2 were referred out of area). Hence there were 49 patients included in our analysis (Figure 1).

### Outcome measure

The primary outcome measure was the proportion of patients achieving clinical and radiological union.

Secondary outcomes measure were clinical variables of significance between those achieving radiological union with EXOGEN and those wherein non-union persisted and considered pathology, patient, and technique related factors as below:

*Pathology related outcomes* – anatomical location of injury; whether the injury was closed or open and if open the classification in accordance with the Gustilo-Anderson grading system; and method of stabilization (conservative verses surgical).

*Patient related outcomes* – the age of patient; the ASA grade; the Charlson co-morbidity index; smoking status; diabetic status.

*Technique-related outcomes* – the delay between injury and definitive stabilization; residual bone gap; the date EXOGEN was applied; the duration of EXOGEN therapy, and whether the union had been achieved.

Additionally, the local practice was observed, and a comparison was made to Bioventus’s standardized guidance, which is provided to clinicians and departments prior to their utilization of the EXOGEN LIPUS device.

### Statistical analysis

Data were analyzed using the software package SPSS 25 (Chicago, IL, USA). Logistic regression was applied to determine the variables predictive of persistent non-union. Significance was accepted at *p* < 0.05.

## Results

### Primary outcome

Following completion of EXOGEN therapy, the radiological union was confirmed in 23 out of 49 (47%) patients.

### Pathology related outcomes

**Figure 2:**
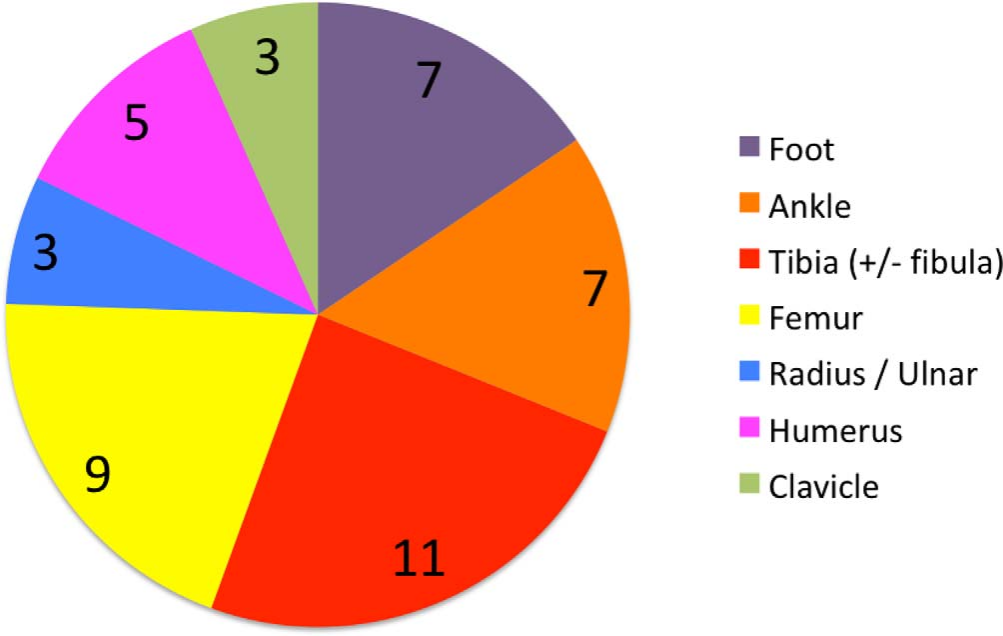
Pie chart demonstrating fracture location for patients included within study.

**Figure 3:**
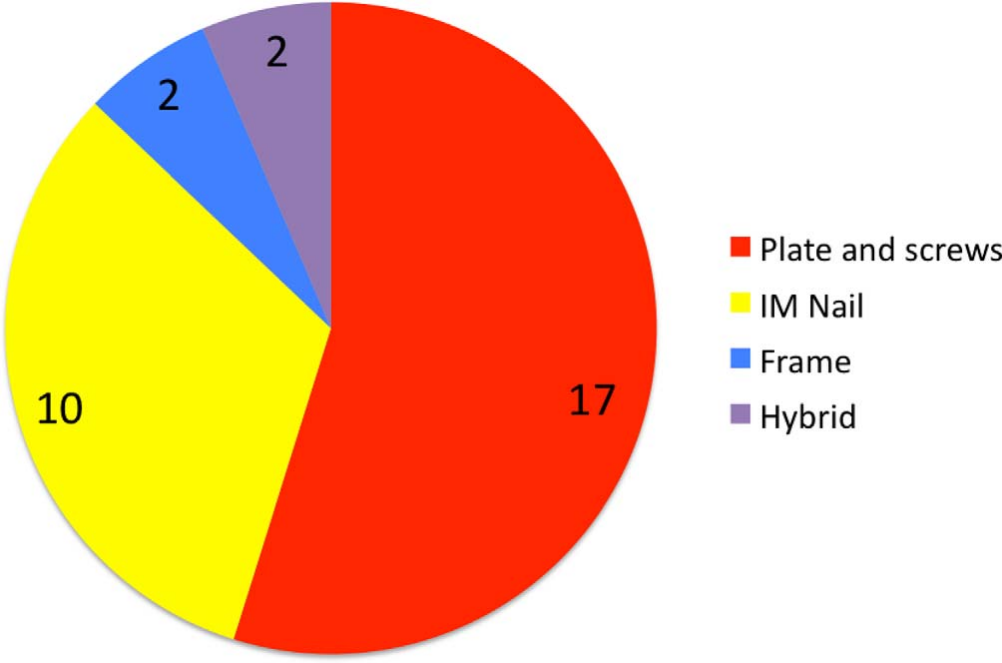
Pie chart demonstrating method of initial surgical stabilization used in an attempt to achieve bone union.

Forty-five patients initially presented with fracture following trauma, with most fractures occurring at the tibia, as shown in Figure 2. Of the 45 with fractures, 35 were closed injuries, and 10 were open. Fourteen of these were managed conservatively, while the rest (31 cases) were managed operatively (Figure 3).

The other subset of patients included were four who had EXOGEN applied following elective surgeries. These consisted of two tibiotalar arthrodesis, one subtalar arthrodesis, and one metatarsophalangeal arthrodesis.

### Patient-related outcomes

Comparison of those patients progressing to union and those patients with persistent non-union revealed no variables of significance in relation to age, ASA grade, comorbidities, smoking, and diabetic status, as evident from Table 1.

**Table 1:** Table with statistical comparison of patient characteristics between union and persistent non-union groups revealing no variables of significance.

Variables predictive of union: logistic regression (*n* = 49)			
Variable	*p*-Value	OR	95% CI
Age	0.193	1.023	0.988–1.060
Open fracture	0.623	0.702	0.171–2.881
ASA grade			
1 (reference group)			
2	0.719	1.273	0.343–4.726
3	0.748	1.273	0.293–5.534
Charison co-morbidity index	0.386	1.184	0.808–1.733
Smoker	0.079	0.324	0.092–1.137
Diabetes	0.313	2.526	0.417–15.297
Surgery	0.130	0.370	0.102–1.339
Time between injury and surgery	0.522	0.989	0.958–1.022
Gap	0.557	0.937	0.754–1.164
Time between injury and ultrasound	0.630	1.000	1.000–1.001
Time between surgery and ultrasound	0.149	1.003	0.999–1.006

### Technique related outcomes

The mean number of days between definitive management and initiation of EXOGEN therapy was 263 days (range 80–753). The mean number of days between initiation of EXOGEN therapy and final radiological review was 132 days (range 56–252). Table 1 further illustrates no significance in achieving union when considering the role of time between injury and stabilization, residual bone gap, and duration of EXOGEN therapy.

### Local practice

Our analysis revealed disparities between recommended practices as 49% of patients had EXOGEN applied before the 9 months falling within NICE’s definition for fracture non-union. In addition, only 4% of patients had their EXOGEN device checked for compliance, deviating from Bioventus’ standardized guidance.

## Discussion

Fracture non-union poses significant consequences to patients, and the risks of a repeat surgery do not come without associated risks, costs, and complications. LIPUS devices, in this case, EXOGEN, provide a cost-effective non-operative solution to promote bone healing in patients with established non-union. Our results show a significant rate of radiological union, yet one below those previously reported.

### Limitations

The primary limiting factor in our study lies within the relatively small sample size, which despite providing valuable insight into the rates of union within local trusts, would reveal a clearer image of the general population with larger patient numbers.

We include non-union of various bones, with differing causative injuries, host factors, initial surgical techniques, and consequently contrasting types of non-union resulting in fluctuating healing capacity irrespective of treatment modality. The nature of non-union, when considering the large number of factors that influence treatment outcome, creates an intrinsic difficulty in studying intervention and generalizing their respective results due to the variance in factors.

At both MFT and SRFT, clinical documentation was of poor quality. To date, EXOGEN therapy has not been closely monitored, outcomes fail to be analyzed and reported. Outcomes monitoring is important to determine if this therapy may prove effective for a specific cohort.

### Radiological union

The confirmed fracture union rate was 47%. If it is assumed that those who did not attend final follow-up and those discharged prior to the final radiological investigation were asymptomatic and hence had achieved union clinically, the fracture union rate increases to 61%. While this finding is positive, in that further surgery was avoided in these patients, it is significantly lower than the union rate supported by EXOGEN at 86% [24], published evidence within the NICE assessment review (Table 2), and that of a previous study published in the North West Deanery, wherein 79% progressed to union [25]. This may be attributed to failings in the correct application of the transducer, follow-up of patients, and compliance to therapy.

**Table 2:** Summary of clinical evidence from published studies used within the NICE assessment review (adapted from Tables 1, 2, & 7 in the External Assessment Centre report).

Study	Study design	Type of long bone fracture	Non-union (NU) or delayed healing (DH)	Mean fracture age (months)	Mean patient age (years)	Healing rate	Mean Healing time
Schofer 2010	RCT	Tibia	DH/NU?	13	43	65% for EXOGEN (33/51)	Not reported
				>9 (*n* = 51/101)	(14–70)	46% for placebo (23/50)	
Lerner 2004	Case series	Femur, tibia, radius/ulna, humerus	DH	6 (range 1–38)	19–48	94% (15/16)	Mean bone union time 75 weeks (34–224)
Jingushi 2007	Case series	Femur, tibia, humerus, radius, ulna	DH/NU	19 (range 3–159)	40 (14–83)	83% (33/40)	Not reported separately for DU and NU
Mayr 2000	Case series	Femur, tibia, fibula, radius, ulna, humerus	DH/NU	3–9 (*n* = 951)	20–71	76% (Humerus 41/54),	125 days (humerus, SD 11.7)
				>9 (*n* = 366)		94% (Radius-ulna 49/52)	115 (Radius-ulna, SD 9.3)
						81% (Ulna 35/43)	130 (Ulna, SD 15.3)
						87% (Femur (85/98)	140 (femur (SD 8.3)
						92% (Tibia 350/380)	138 (tibia SD 4.5)
						96% (Fibula, 26/27)	113 (fibula, 9.6).
						Total 90% (586/654)	Mean 4.4 months
Gebauer 2005	Case series	Tibia, fibula, femur, humerus, radius, ulna	NU	>8	23–86	90% (long bones, 46/51)	178 days (86-375) for long bone fractures
						85% (all fractures, 57/67)	168 days for all fractures.
Nolte 2001	Case series	Humerus, radius, ulna, femur, tibia, fibula	NU	15 (range 6–34)	18–90	100% (Tibia-fibula, 10/10)	144 days (Tibia-tibia/fibula)
				<9 (n = 5/21)		80% (Femur, 4/5)	185 (femur)
						80% (Radius-ulna, 4/5)	
						100% (other long bones, 2/2)	139 (radius-radius/ulna, 4/5)
						Duration was 2 years.	153 (other long bones, 2/2)
							
Romano 1999	Case series	Tibia, humerus, femur	NU (septic)	8–30 < 9 (*n* = 1/13)	28–78	62% (8/13 tibia, humerus, femur)	95–181 days (3 still in treatment at time of report)

### Cost-effectiveness

NICE has also reviewed literature specific to EXOGEN, reporting that clinical evidence supports the use of EXOGEN to facilitate healing in patients with established non-union of long bones (persisting over 9 months) and that this is associated with a cost saving of £2407 per patient, through avoiding surgery. It is important to consider the additional cost incurred for patients with persistent non-union after EXOGEN therapy. In our experience, 69.2% of patients were referred for further surgery, 19.2% were prescribed further EXOGEN therapy, and 7.7% were unfit for surgery.

EXOGEN therapy comes at an additional cost for trusts, with departments submitting an individual funding application for each case. The application necessitates a description of the patient’s presentation, current clinical situation, and explanation of why EXOGEN is in the patient’s best interest. Freedom of information requests has revealed that between 2017–2019 purchase of EXOGEN devices cost the MFT £44,000 and the SRFT £174,244. When considering the cost of personnel and clinic time, the total cost will be considerably higher. It is critical that EXOGEN is utilized correctly to ensure maximum efficacy and hence cost-effectiveness.

### Common pitfalls in local practice

Our analysis revealed that 49% of patients had EXOGEN applied before the 9 months recommended by NICE as the definition for fracture non-union [23]. NICE reports some evidence of improved healing in those with the delayed union (3 months post-injury/fixation), yet the uncertainties with which bone healing progresses without adjunctive treatment between 3–9 months after fracture and whether or not surgery would be necessary persist. These uncertainties could mean that instigating EXOGEN therapy earlier results in greater expense than current management protocols [26]. The chronology of the patient’s presentation should be accurately documented when making a funding application and requires vetting before funding is granted.

Appendix A (Table A1) demonstrates Bioventus’ screening tool for their EXOGEN device. This screening tool reinforces the importance of compliance to therapy however, there remains a lack of information on the correct application of the transducer. One key limitation of EXOGEN treatment is the size of the transducer. Bioventus advises that the transducer’s size limits its therapeutic area to 3.88 cm^2^ [27]. Despite the ability of ultrasound waves to pass through some 20 cm of soft tissue to reach the underlying bone, the narrow therapeutic diameter necessitates accurate placement of the transducer over the site of the non-union. Achieving accurate transducer placement upon every application may prove difficult for some patients.

Recommendations include marking the skin with radio-opaque markers and obtaining X-rays. The markers may take the form of a series of paper clips taped to the skin. The markers are to remain in situ until the X-rays have been reviewed. As such, they may be used as reference points to determine the correct site for transducer placement. Radiologists and radiographers must be informed to obtain X-rays with the patient positioned as they would be for the delivery of EXOGEN therapy at home. This is particularly important for bones with large soft tissue coverage (e.g., femur and humerus) as the patient’s position can greatly influence the surface anatomy. Using a skin marker, the correct application site must be emphasized to the patient when educating them on the use of the EXOGEN device.

Follow-up must be standardized, and the authors recommend 8 weekly intervals. At each follow-up visit, the site of transducer placement must be re-assessed. This necessitates a set of repeat X-rays with the patient citing another set of radiopaque markers at the site of transducer application. In this way, the clinician can determine if the non-union site remains within the therapeutic area of the transducer. This is important as the transducer position may migrate with repeated applications by the patient.

**Figure 4:**
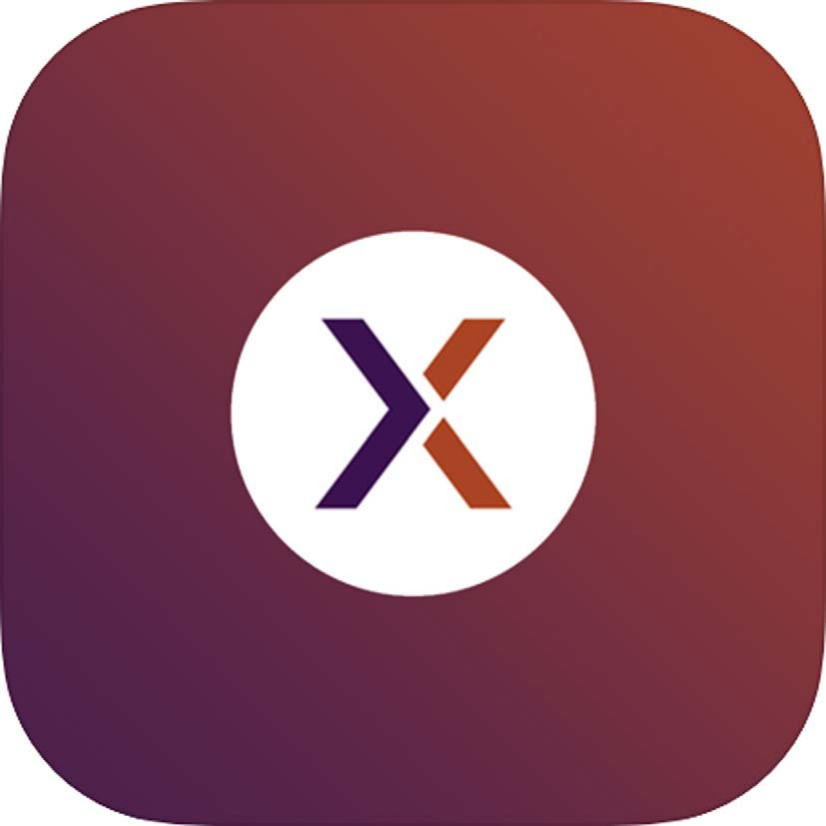
Exogen connects app icon.

The literature advises that compliance to EXOGEN therapy can be poor, with one study reporting a compliance rate of just 43% [28]. Patients should be routinely assessed for their ability to comply with therapy. This forms part of the funding application. Accurate completion of this application will help identify the appropriate patient cohort. The EXOGEN device routinely monitors compliance, yet this was documented in just 4% of patients included in our study. Poor compliance could explain the discrepancy between our outcomes and that of previously published studies. To investigate this, details of compliance should be routinely recorded at each follow-up appointment. Patients should be informed to download the free EXOGEN Connects app (Figure 4). This app allows the patient to save an image of where their transducer should be fitted and will send them daily reminders to perform their EXOGEN treatment. If compliance remains poor, further EXOGEN therapy for persisting non-union is not advised.

**Figure B1.**
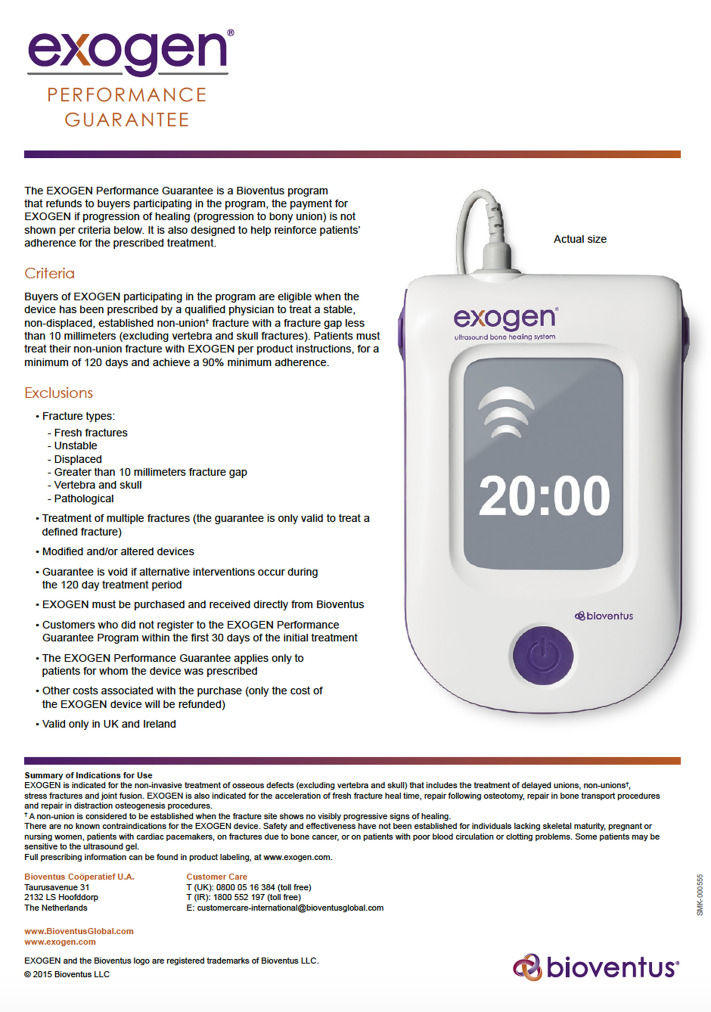
Bioventus’ union or money back guarantee.

Local discussion may help identify factors that may be addressed to improve efficacy. Bioventus offers a union or money-back guarantee, and provisions for this guarantee to be valid are detailed in Appendix B (Fig. B1). Failure to meet Bioventus’ recommendations will invariably cost both hospitals and Trusts. The authors advise that confirmation of a valid guarantee is confirmed prior to commencement of therapy. Some fractures have high mobility, and difficulties in stabilizing these fractures can invalidate the guarantee. This is true for humeral fractures, wherein the rate of successful healing with EXOGEN is the lowest.

### Exogen vs. alternatives

Other LIPUS system alternatives are available within the European Union (EU), with various studies supporting their use. Exogen (Bioventus) has the most available clinical data with 32 studies supporting its use, followed by Melmak (Melmak) with two studies, then FASTerapia (Igea) with one study, and lastly, LIPUS (N-Dis GbR) with none [27]. The sparse clinical data on Exogen alternatives limit our ability to create any comparisons regarding efficacy and cost-effectiveness at present.

Making conclusions on the efficacy of EXOGEN for patients presenting with non-union to the MFT and the SRFT will necessitate a review of current practice, education of clinical personnel and patients, together with prospective data collection and local outcomes reporting.

## Conclusion

Failure to utilize the EXOGEN device correctly and monitor compliance will impact outcomes. This therapy comes at an additional cost for Trusts, but failure to achieve bone union may result in the patient being exposed to the risks of further surgical intervention. Outlined is a standardized approach, which avoids the main pitfalls in prescribing and monitoring EXOGEN LIPUS therapy.

## Institutional approval

Institutional approval was not required.

## Appendix A

**Table A1:** EXOGEN device suitability screening tool to be used to assess patient suitability prior to commencing EXOGEN and assessing for successful completion post-treatment.

EXOGEN^®^ ultrasound bone healing system used for the management of long bone non union fractures					
**Patient NHS No.**		**Trust:**		**GP Name:**	
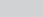
**Patient Hospital Number:**		**Consultant Making Request:**		**GP code/Practice code:**	
**Patient initials**					
**Date of birth**		//		**GP Post code:**	
**Please confirm the following**					
The patient is over 18 years old.				Yes	No
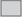
The patient has a non-union fracture or arthrodesis for > 9 months The bones are well aligned, stable and the inter-fragment gap is < 10 mm. Date of fracture // and type and location of long bone fracture				Yes	No
The patient has been screened and referred by a Consultant Radiologist/Consultant Orthopaedic Surgeon following review on at least two occasions at least 4 weeks apart to allow examination of serial X-rays. The patient has received a further assessment in a non-union clinic by surgeon with expertise of dealing with non-union of long bones; appropriateness of EXOGEN^®^ has been determined through agreement of two specialist non-union Consultants.				Yes	No
The patient has been counselled and has the ability to comply with usage protocol and criteria in line with the EXOGEN International* Performance Program which includes a 90% minimum adherence to the treatment regimen for minimum of 120 days The patient is registered on the EXOGEN International* Performance Program. Purchaser Code				Yes	No
**For treatment failures, the provider will ensure that a reimbursement is obtained in accordance with the manufacturers “money back guarantee” arrangement; the CCG will not fund these patients.**					
**I confirm that the patient meets the criteria for treatment**			**I confirm that the patient meets the criteria for treatment**		
Name of consultant:			Signature (or email confirmation) by Department Service Manager (or nominated deputy)		
Signature:			Name:		
Date: //			Signature: Date: //		
**Section below to be submitted on completion of treatment**					
Treatment was successful				Yes	No
If no, seek reimbursement from the manufacturer Cost of EXOGEN^®^ claimed £					
Time to heal/fail (weeks) Date final assessment //					
**I confirm that the patient meets the criteria for treatment**			**I confirm that the patient meets the criteria for treatment**		
Name of consultant:			Signature (or email confirmation) by Department Service Manager (or nominated deputy)		
Signature:			Name:		
Date: //			Signature: Date: //		
